# A Systematic Study on the Structural and Optical Properties of Vertically Aligned Zinc Oxide Nanorods Grown by High Pressure Assisted Pulsed Laser Deposition Technique

**DOI:** 10.3390/nano8020062

**Published:** 2018-01-25

**Authors:** Priyanka Karnati, Ariful Haque, M. F. N. Taufique, Kartik Ghosh

**Affiliations:** 1 Department of Physics, Astronomy and Materials Science and Center for Applied Science and Engineering, Missouri State University, Springfield, MO 65897, USA; ahaque@ncsu.edu or ariful114@live.missouristate.edu (A.H.); m.taufique@wsu.edu (M.F.N.T.); kartikghosh@missouristate.edu (K.G.); 2 Department of Materials Science and Engineering, Ohio State University, Columbus, OH 43210, USA; 3 Department of Materials Science and Engineering, North Carolina State University, Raleigh, NC 27695, USA; 4 School of Mechanical and Materials Engineering, Washington State University, Pullman, WA 99163, USA

**Keywords:** ZnO nanostructures, Raman spectroscopy, oxide semiconductors, optoelectronics, photoluminescence, defects, pulsed laser deposition

## Abstract

In this study, we synthesize high quality vertically aligned ZnO (VAZO) nanorods on silicon, sapphire, and indium tin oxide (ITO) substrates by using pulsed laser deposition (PLD) technique at high growth pressure (0.3 Torr). Systematic changes in structural and optical properties of VAZO nanorods are studied by varying the substrate temperature (500–600 °C) and number of pulsed laser shots during the deposition. ZnO nanoparticles deposited at high pressure act as nucleation sites, eliminating requirement of catalyst to fabricate VAZO nanorods. Two sharp ZnO peaks with high intensity correspond to the (0002) and (0004) planes in X-ray diffraction pattern confirm the growth of ZnO nanorods, oriented along the *c*-axis. Scanning Electron Microscopy (SEM) images indicate a regular arrangement of vertically aligned hexagonal closed pack nano-structures of ZnO. The vertical alignment of ZnO nanorods is also supported by the presence of E_2_ (high) and A_1_ (LO) modes in Raman spectra. We can tune the diameter of VAZO nanorods by changing growth temperature and annealing environments. Photoluminescence spectroscopy illustrates reduction in defect level peak intensities with increase in diameter of VAZO nanorods. This study signifies that high pressure PLD technique can be used more efficiently for controlled and efficient growth of VAZO nanorods on different substrates.

## 1. Introduction

ZnO has a direct band gap of 3.37 eV and exciton binding energy of 60 meV at room temperature, having device applications [1,2]. It is transparent in the visible wavelength range and has optoelectronic applications such as light emitting diodes (LEDs), transparent electrodes, and ultraviolet (UV) lasers [3,4]. ZnO nanostructures with different morphologies have drawn interest over the years, as having potential electronic applications and nano-structuring can improve performances of already existing devices by increasing the surface or interface area while maintaining the constant volume [5,6]. Vertically aligned zinc oxide (VAZO) nanorods are potentially useful for vertical device fabrication including light emitting diodes, solar cells, and nano piezoelectronics [7,8]. Numerous growth processes, such as chemical vapor deposition [9,10] and various forms of physical vapor deposition techniques [11,12], have been used to synthesize VAZO nanorods in presence of a catalyst at high temperature and following a vapor-liquid-solid (VLS) mechanism. However, it has been a big challenge to find a controlled technique to grow well aligned ZnO nanorods. To gain control over the morphology, density, and the orientation of the grown nanostructures, the essential growth and processing steps of ZnO nanostructure formation need to be understood. In pulsed laser deposition (PLD) technique, nanostructure growth via vapor-solid (VS) mechanism can be initiated by the gas phase formation of nanoparticles at high pressures. The nanoparticles deposited on the substrate act as nucleation sites and promote nanostructure growth [13]. In general, VS-grown nanostructures are catalyst free and no additional steps are required for the removal of catalyst particles at the tips of the nanostructures in order to fabricate efficient devices. The dimensions of the grown nanostructures, however, depend only on the growth conditions and if employed on the nucleation layer. Therefore, the growth of nanostructures by the VS mechanism as well as their locations and densities are often more difficult to control than nanostructure formation by a catalyst. The present work involves growth of VAZO nanostructures without a catalyst, which follows VS growth mechanism. Annealing studies were performed to systematically analyze the properties of the VAZO nanorods. Different parameters such as influence of substrates, growth temperature, number of pulsed laser shots, and annealing temperature and environment—i.e., oxygen and forming gas (95% Ar and 5% H_2_)—were considered to study the properties of VAZO nanorods. Moreover, the growth mechanism has been studied by varying the substrates, which plays a major role in the formation of the nanorods and also on the alignment. Varying the growth temperature and number of pulsed laser shots help to study the variation in diameter, length, and structural properties, such as crystallinity and defects, in the structure of the VAZO nanorods.

## 2. Results

### 2.1. Scanning Electron Microscope (SEM) Analysis

**Figure 1:**
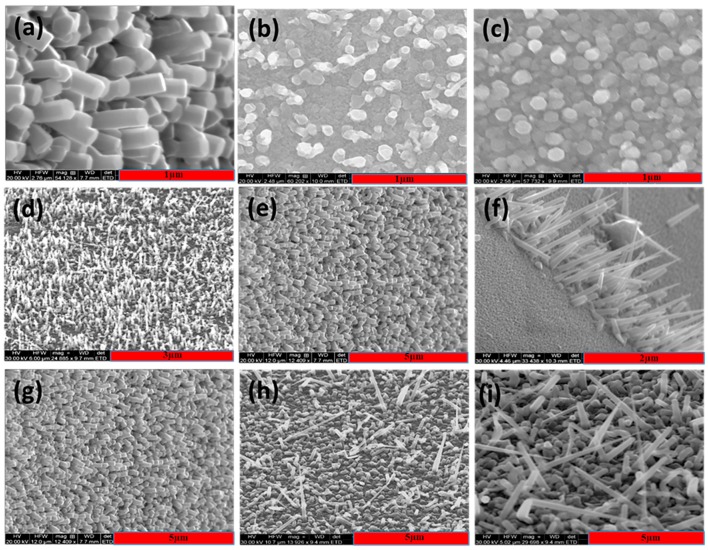
SEM Images of ZnO nanorods (**a**) Vertically aligned on silicon substrate (60° titled view), (**b**) Sapphire substrate (top view), (**c**) Indium tin oxide (ITO) substrate (top view), SEM images of vertically aligned ZnO nano rods (5000 shots) grown at different temperatures on silicon substrate (60° titled view) (**d**) 500 °C, (**e**) 550 °C, (**f**) 600 °C and SEM images of the ZnO nanorods by varying the number of pulsed laser shots at 550 °C temperature (**g**) 5000, (**h**) 10,000, and (**i**) 15,000.

Figure 1a–c represent the field emission scanning electron microscope (FESEM) images of the ZnO nanorods on silicon, sapphire, and indium tin oxide (ITO) substrates. Wurtzite structure of ZnO can be confirmed from the FESEM images and the diameter of 1D nanostructure was measured to be in between 300–500 nm. The vertical alignment of these nanorods is high when grown on the silicon substrate, which mainly depends on the growth mechanism and surface energy of the Si substrate [14]. Figure 1d–f represents the SEM images of the VAZO nanorods grown on Si substrate at different temperatures (500, 550, and 600 °C). The variation in the diameter of these nanorods is evident and ranging from 50–500 nm were calculated by using ImageJ software. The increase in the substrate temperature resulted in an increase in the diameter of the rods, which is mainly dependent on the growth mechanism and stress between the substrate and nanorods formed [15]. From the SEM images in Figure 1g–i, we observe an increase in length of the nanorods, varying from 400 nm^–1^ µm, with increasing number of pulsed laser shots. From these SEM images, it is also evident that the nanorods tend to bend as the number of pulsed laser shots increases, which mainly depends on the stability of the nanorods [16]. The diameter and length of the nanorods has been varied as we change the process parameters. The average diameter and length of the nanorods are shown in Table 1.

### 2.2. X-Ray Diffraction Analysis (XRD) 

**Figure 2:**
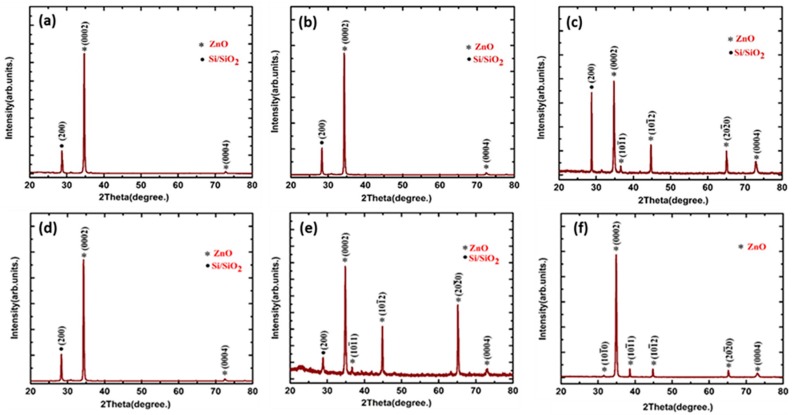
XRD spectrum of VAZO nanorods grown on Si substrate at (**a**) 500 °C; (**b**) 550 °C; (**c**) 600 °C and XRD spectrum of VAZO nanorods grown on Si substrate at 550 °C by varying the number of shots (**d**) 5000, (**e**) 10,000, (**f**) 15,000.

Figure 2a–c represent the X-ray diffraction pattern of ZnO nanorods on Si substrate. In addition to the substrate peak, VAZO nanostructures show strong peaks corresponding to (0002) and (0004) planes. The strong peak associated with (0002) plane implies that the ZnO nanostructures were preferentially oriented along the *c*-axis [17]. The preferred orientation of ZnO nanostructures along (0002) also indicates that the as grown nanostructures have good epitaxial orientation with the Si substrate, which can also be verified through SEM images shown previously. From Figure 2a,b it is evident that the nanorods deposited at 500 °C and 550 °C are preferentially oriented along the (0002) plane.

Figure 2c represents the XRD pattern of sample deposited at 600 °C confirming the presence of other ZnO planes. This implies that the orientation of ZnO nanorods has been changed with increasing deposition temperature in the PLD system. Figure 2d–f represents the XRD spectrum of ZnO samples grown by varying the number of pulsed laser shots from 5000 to 15,000 at 550 °C. The increased number of pulsed laser shots led to the formation of bulk ZnO, which is confirmed through XRD pattern. Full Width Half Maxima (FWHM) of (0002) plane of ZnO samples has been calculated by using Labspec 5 software, which helps in determining the crystallinity in the samples. R. R. Reeber measured lattice constants of ZnO wurtzite structure at room temperature, values of *c*, *a* being 5.2075 Å, 3.25 Å, respectively, and resulting $\frac{c}{a}$ ratio 1.633 [18]. Measured FWHM value of (0002) plane, interplanar spacing (*d*), lattice parameters (*c* and *a*), and the $\frac{c}{a}$ ratio calculated for ZnO nanorod samples deposited on Si substrate are shown in Table 2.

The following formula was used for calculating the lattice parameters.

(1) $$\frac{1}{d^{2}}=\frac{4}{3}\;\left(\frac{h^{2}+hk+l^{2}}{a^{2}}\right)+\frac{l^{2}}{c^{2}}$$

### 2.3. Raman Spectra Analysis

**Figure 3:**
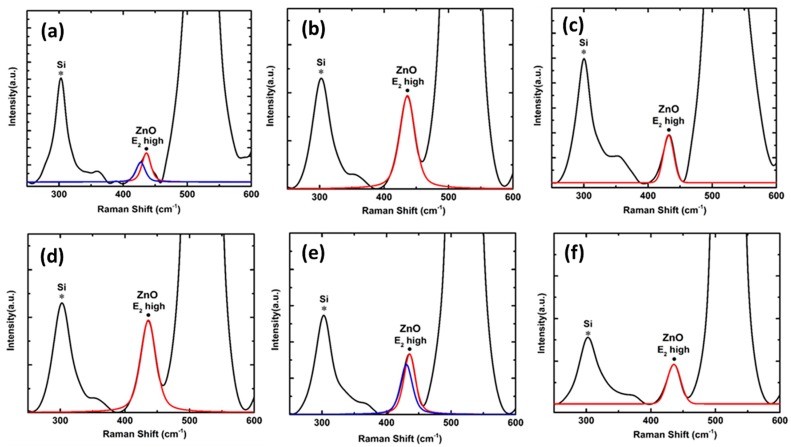
Raman spectra of ZnO nanorods at different temperatures (**a**) 500 °C, (**b**) 550 °C, (**c**) 600 °C and Raman spectra of ZnO nanorods grown at 550 °C by varying the number of pulsed laser shots (**d**) 5000, (**e**) 10,000, (**f**) 15,000.

**Figure 4:**
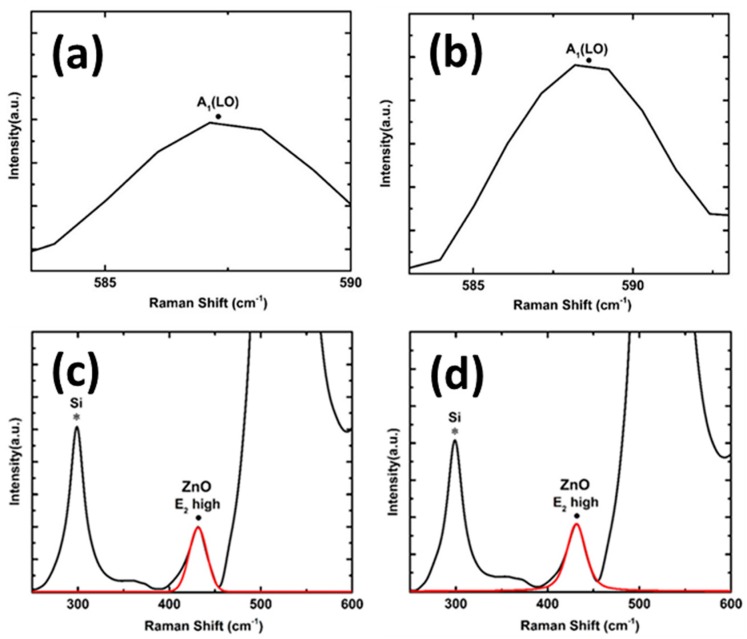
A_1_ (LO) mode in Raman spectra of aligned ZnO nanorods at (**a**) 500 °C (**b**) 550 °C and E_2_ high mode of aligned ZnO nanorods grown at 500 °C, annealed in (**c**) O_2_ atmosphere (**d**) H_2_ atmosphere.

Figure 3a–c represents the Raman spectra of the VAZO nanorods grown on Si substrates at different temperatures. Wurtzite ZnO belongs to$\;C_{6\upsilon }^{4}$ (*P 6*_3_ *mc*) space group. The primitive cell includes two formula units with all atoms occupying 2b sites of symmetry. According to group theory, wurtzite ZnO structure is expected to have A_1_ (*z*) + 2B_1_ + E_1_ (*x, y*) + 2E_2_ optical phonon modes at the Г point of the Brillouin zone [19]. As a result, A_1_ and E_1_ phonon modes are infrared and Raman active. Raman modes that can be observed in the spectrum mainly depend on the Raman selection rules and geometry employed to attain the spectra. Backscattering (z) geometry was used to perform the Raman spectroscopy. The *c*-axis of wurtzite ZnO structure is along the *z* direction, hence, Raman peaks of A_1_ (LO) and E_2_ (high) are allowed according to Raman selection rules [20]. Raman spectra of different ZnO nanorod samples shown in Figure 3 confirm the presence of E_2_ high mode. The peak position and FWHM of the Raman E_2_ high mode of the ZnO nanorods were determined by LabSpec 5 software using Lorentzian function. The position of E_2_ high peak varies from 436.16 cm^−1^ to 436.24 cm^−1^ for samples grown at 500 °C and 550 °C, respectively. Since E_2_ high mode is more sensitive to stress, compressive stresses are responsible for the shift to a higher value [21] when the deposition temperature is increased leading to increase in diameter of VAZO nanorods. The presence of quasi modes can also be seen in the Raman spectra of Figure 3b, which is dependent on bending and alignment of the rods [22]. The amount of stress developed in the VAZO nanorod samples is less compared to the ZnO thin films, which may be due to the relaxation effect of the ZnO nanorods. Figure 3d–f represents the Raman spectra of ZnO nanorod samples grown at 550 °C with varying number of pulsed laser shots from 5000 to 15,000. The FWHM of the E_2_ high phonon mode changes with increasing number of pulsed laser shots. This is due to the dependence of the FWHM of this peak on the crystallinity of ZnO nanorods. FWHM of the ZnO sample grown by 15,000 pulsed laser shots increases and peak position matches with that of ZnO thin film. A_1_ (LO) mode can only be seen in Raman spectra of aligned ZnO nanorod samples as per Raman selection rules [23]. Figure 4a,b represent A_1_ (LO) mode present in aligned ZnO nanorod samples. This mode has only been seen in samples grown at 500 °C and 550 °C and alignment of ZnO nanorods can be verified through high resolution SEM images. The peak positions of E_2_ high phonon mode are represented in Table 3.

Annealing experiments were performed in both oxygen and hydrogen medium at 500 °C for the ZnO sample grown at the same temperature on Si substrate. A pressure of 0.3 mbar was maintained during the annealing process. Figure 4c,d represents Raman spectra of samples annealed in oxygen and hydrogen environments, respectively. A red shift in E_2_ high phonon mode was observed when compared to the peak position of E_2_ high phonon mode of ZnO nanorods sample before annealing. The peak position of E_2_ high phonon mode for samples annealed in oxygen atmosphere and hydrogen atmosphere were found to be at 431.36 cm^−1^ and 431.05 cm^−1^, respectively. Peak positions in both samples are in coherence with ZnO thin film, which implies that the alignment of the ZnO nanorods has been changed. A decrease in the FWHM of this peak was observed after the annealing treatment. It can also be inferred that a sharper E_2_ high mode signifies a greater crystallinity of ZnO nanorods. Crystallinity of ZnO nanorod samples can also be confirmed from photoluminescence studies by observing the change in defect levels.

### 2.4. Photoluminescence (PL) Spectra Analysis

**Figure 5:**
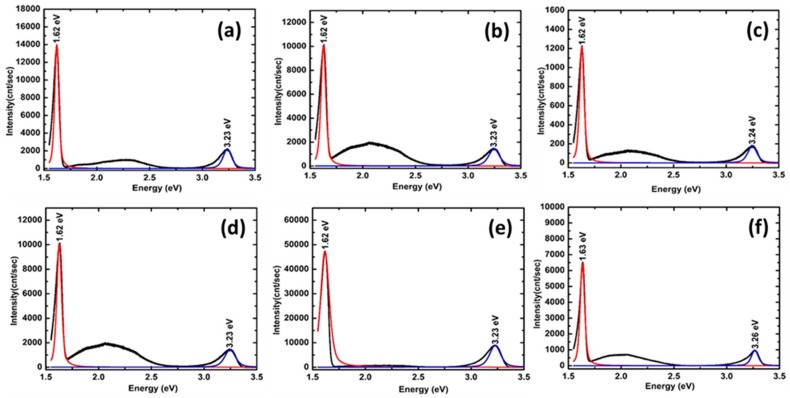
PL spectra of ZnO nanorods grown by using 5000 pulsed laser shots at different temperatures (**a**) 500 °C, (**b**) 550 °C, (**c**) 600 °C and PL spectra of ZnO nanorods grown at 550 °C by varying the number of pulse laser shots (**d**) 5000, (**e**) 10,000, (**f**) 15,000.

Optical properties of the VAZO nanorods were investigated by performing the PL spectroscopy. Figure 5a–c represents PL spectra of ZnO nanorods at temperatures 500, 550, and 600 °C, respectively. All PL spectra show a luminescence emission in-between 3.23 eV and 3.24 eV, which is due to direct recombination of the excitons [24]. Appearance of one UV peak for nanorods is an indication of uniform distribution of the rod size [25]. ZnO samples in Figure 5a,c grown at 500 °C and 600 °C, respectively, show a wide and strong deep level emission around 2.08 eV. This orange emission has been found in oxygen rich ZnO films grown by PLD process and is attributed to oxygen interstitial defects [26,27]. ZnO samples were grown in highly oxygen rich atmosphere and at high temperature; hence, there is a possibility for the formation of oxygen interstitials in the structures. In the ZnO sample grown at 550 °C, a shift in the deep-level green emission at ~2.23 eV has been observed as shown in Figure 5b. This occurs due to the structural defects such as oxygen vacancies or zinc interstitials [28]. With increase in growth temperature from 500 °C to 600 °C, an increase in size of the VAZO nanorods was observed from SEM image analysis. For ZnO nanostructures the effect of surface status on the PL intensity must be carefully considered [29]. Band bending creates an electron depletion layer around the ZnO rod surface, which is more than 100 nm. This layer plays a key role as the photoemissions occur deep from the surface [30]. Therefore, as the ZnO nanorods diameter is increasing, where the depletion layer is not so important, the oxygen vacancies lead to stronger green emission compared to the nanorods with smaller diameter reducing the green emission intensity [11]. With an increase in the growth temperature (600 °C), in case of Figure 5c, we can see a decrease in deep level emission intensity because of the improved crystallinity in the sample.

**Figure 6:**
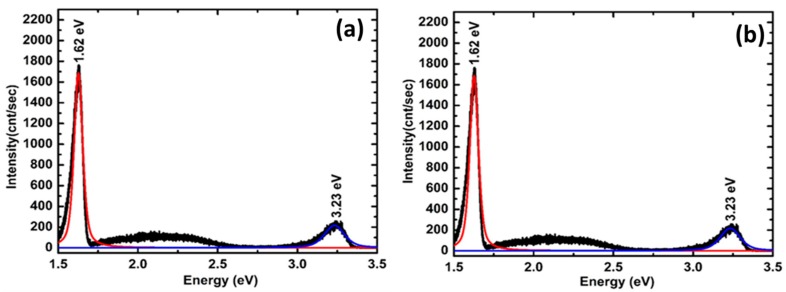
PL spectra of ZnO nanorods grown at 500 °C and annealed in (**a**) O_2_ atmosphere (**b**) H_2_ atmosphere.

Figure 5d–f represents PL spectra of ZnO nanorods samples grown by 5000, 10,000 and 15,000 pulsed laser shots, respectively. Length of ZnO nanorods has been increased with the number of pulsed laser shots, which was verified from SEM images. A reduction in the green emission level intensity was observed in Figure 5e compared to that in Figure 5d. When the number of pulsed laser shots during the deposition was increased to 15,000, bending of rods can be seen and the tendency to form thin films is evident from SEM image. The tendency to form thin film can be seen in the presence of orange-red defect level, which is mainly due to the presence of oxygen interstitials. This defect level is visible in the PL spectra presented in Figure 5f. To confirm the origin of defect peaks from the PL analysis, VAZO nanorods grown at 500 °C were annealed in both oxidizing and reducing atmosphere. After annealing for 30 min in oxygen atmosphere at 500 °C, the defect peak intensity related to the oxygen interstitial is reduced, which can be observed from Figure 6a. Improved crystallinity of VAZO nanorods can be confirmed after annealing in oxygen atmosphere. Reduced oxygen interstitial concentration can be attributed to oxygen desorption from ZnO nanorod structure, which occurs due to the annealing at high temperature (500 °C) [31]. Similarly, annealing of ZnO nanorods was carried out in reducing environment (H_2_) at 500 °C and the associated PL spectrum is shown in Figure 5b. Reduction of peak intensity of oxygen interstitials was observed in PL spectra. Due to large surface area and small diameter of ZnO nanorods, hydrogen diffuses readily into the crystal rods to further reduce oxygen present in ZnO crystal, thus reducing the oxygen interstitials [32]. The shift in defect peaks by changing the process parameters has been listed in the Table 4.

## 3. Discussion

Several parameters are responsible for the change in alignment, structural and optical properties of ZnO nanorods grown by PLD technique. Among these substrate types, growth temperature, number of pulsed laser shots, and annealing environment play important roles. XRD analysis explains about the crystallinity and the preferred orientation of the ZnO nanorods, which is not sufficient to confirm the vertical alignment of the rods. SEM and Raman analysis help to verify the vertical alignment of ZnO nanorods. SEM analysis helps in determining the average length and diameter of the ZnO nanorods. The diameter varies from 50 nm to 500 nm as the growth parameters are changed. Vertical alignment is visible from SEM images. The presence of E_2_ high and A_1_ (LO) modes in Raman spectra also supports this. Alignment is also dependent on the substrate used, which follows Volmer-Weber model for formation of the ZnO nanorods. Growth of ZnO nanorods follows VS mechanism, i.e., formation of the nanorods without a catalyst through PLD technique.

ZnO nanoparticles ablated from the target at high pressure act as nucleation sites and help in the formation of nanorods. Alignment of ZnO nanorods, which mainly depends on surface energies, was found to be more efficient on Si substrate. Smaller FWHM of the E_2_ high phonon mode signifies better alignment of the ZnO nanorods. Change in growth temperature during the pulsed laser deposition of ZnO samples helped in determining apt parameters for fabricating VAZO nanorods with desirable properties. An increase in the diameter of ZnO nanorods was observed as the temperature was increased, which is evident from the SEM analysis and form the shift in E_2_ high mode in Raman spectroscopy. The change in defect levels was seen in PL spectra due to increase in diameter of VAZO nanorods. Band bending and formation of the depletion layer played key roles in shift of defect levels of VAZO nanorods. A change in length of the rods was detected from SEM images as the number of pulsed laser shots was changed. As the number of laser shots was increased from 5000 to 15,000, a shift in E_2_ high mode was detected and absence of A_1_ (LO) mode, which mainly explains about the vertical alignment of the ZnO nanorods, was observed. In PL spectra, a change in UV band emission has been observed for ZnO nanorods sample grown at 15,000 shots, which coincides with bulk ZnO. The reduced intensity of defect levels also explains the crystallinity of the ZnO sample. 

**Figure 7:**
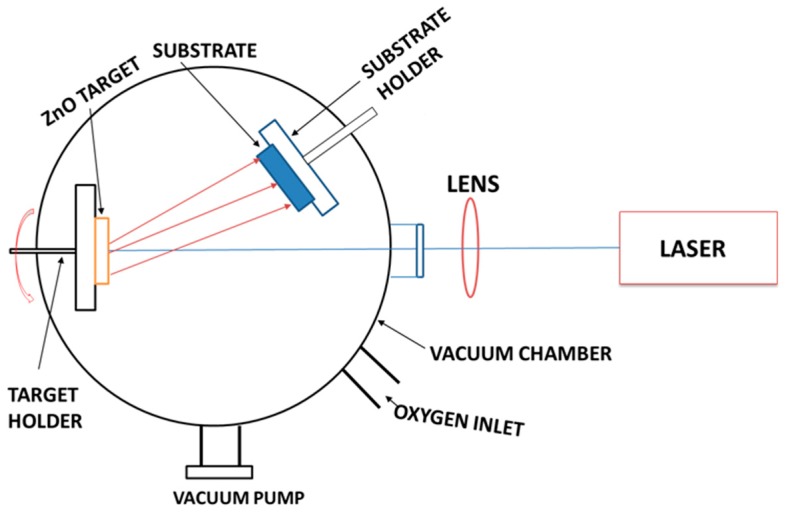
Pulsed laser deposition (PLD) setup used for synthesis of vertically aligned ZnO nanorods (VAZO).

**Figure 8:**
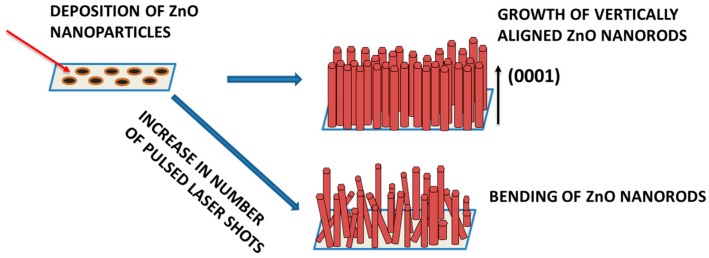
Schematic diagram of growth of vertically aligned ZnO nanorods (VAZO) by using high pressure assisted pulse laser deposition (PLD) process.

Annealing treatment was performed to analyze the defect levels of VAZO nanorods in both oxidizing and reducing atmosphere. Annealing in the presence of oxygen atmosphere led to a decrease in intensity of defect level as seen in PL spectra and a shift in E_2_ high Raman mode. Absence of A_1_ (LO) phonon mode and peak position of E_2_ high mode coincides with the bulk ZnO, which proves that the alignment of nanorods has been changed. Decreased intensity of the defect levels associated with the oxygen interstitials can also be attributed to the temperature (500 °C) used during the annealing treatment, which helped the oxygen atoms to move away from the ZnO wurtzite structure. Annealing in the presence of reducing atmosphere led to reduced intensity of the defect levels. Large surface area and smaller diameter of VAZO rods might help hydrogen atoms to diffuse easily through the surface and reduce the oxygen interstitials present in the structure. A shift in the Raman mode is also observed, which signifies better alignment of the ZnO nanorods. Figure 7 represents Pulse laser deposition (PLD) setup used for synthesis of vertically aligned ZnO nanorods. Figure 8 represents the schematic diagram of growth of VAZO nano rods starting from the deposition of ZnO nanoparticles during initial stage, and then formation of VAZO nanorods after entire deposition process. Bending of the VAZO nanorods has also been shown as we increase in number of pulsed laser shots.

## 4. Materials and Methods

VAZO nanorods were synthesized from a highly dense and pure ZnO target by using PLD technique (Excel Instrument, PLD-STD-18). Laser source used was Lambda Physik, COMPEX201 high energy UV KrF excimer laser. The fabricated nanorods were grown at an average laser energy density in between 3–4 J/cm^2^. During the deposition process, the pulsed laser frequency was maintained at 10 Hz with a pulse duration of 20 ns. The laser was focused on to a 1.9 cm diameter ZnO target prepared by 99.9% pure micron sized ZnO powder. The distance between the target and the substrate was ~3 cm during deposition of VAZO nanorods. Figure 7 represents the pulse laser deposition (PLD) setup used for synthesis of vertically aligned ZnO nanorods. The substrates used were *n*-doped 400 µm Si (111) purchased from Siltronic Ag, ITO coated on glass and Al_2_O_3_ substrates purchased from Aldrich (St. Louis, MO, USA). The chamber was maintained at high pressure, 0.3 mbar of oxygen gas throughout the deposition and the growth temperature was changed in between 500 °C and 600 °C. After the completion of the deposition, the VAZO nanorod samples were cooled down in the same chamber pressure as maintained before. Annealing studies of VAZO nanorods has been done in oxygen and forming gas (95% Ar and 5% H_2_) atmospheres at a pressure of 0.3 mbar and a temperature of 500 °C for 30 min. FEI Quanta 200 S field emission secondary electron microscope (FESEM, Waltham, MA, USA) was used to collect high resolution images of VAZO nanorods. As the nanorods were vertically aligned, it was difficult to collect the images under normal operating conditions. Better quality images were obtained by tilting the specimen stage at an angle of 60°. The diameter and length of the VAZO nanorods were obtained using FESEM images through ImageJ software. We used Bruker D8 Discover X-ray diffractometer (Woodlands, TX, USA) coupled with a Cu Kα emission source (λ = 1.518 Å) to investigate the crystal structure of the VAZO nanorods. Operating current and voltage was maintained at 40 mA and 40 KV, respectively, during the XRD measurements. The 2θ value was maintained in the range between 20° and 80°. We used a Horiba Labram PL-Raman system (Irvine, CA, USA) for the Raman and photoluminescence (PL) measurements on the VAZO nanorods. A green laser with a wavelength of 532 nm was used to perform Raman spectroscopy on the as grown VAZO nanorods. Calibration was performed with a standard Si sample prior to all the measurements. PL spectroscopy was performed by using a 325 nm wavelength laser source. Peak fitting and analysis of the Raman and PL spectra were conducted by using Labspec 5 software.

## 5. Conclusions

VAZO nanorods on Si substrate have been fabricated by a catalyst free high pressure PLD technique. Effects of the substrate type, growth temperature and number of pulsed laser shots have been studied to analyze the crystalline properties of the VAZO nanorods. Factors responsible for alignment of ZnO nanorods have been investigated, which mainly depend on growth parameters. Temperature and number of pulsed laser shots during the deposition play key roles in the alignment of the ZnO rods, which was verified from the E_2_ high mode of VAZO nanorod structures. A way to tune the defect levels is established by changing the diameter of the nanorods, which was performed by changing the growth temperature. Changes in the UV band emissions have been achieved by increasing the number of pulsed laser shots. Annealing studies helped in analyzing defect levels with the help of PL spectroscopy. PL spectroscopy also helped to interpret the reasons behind the observed decrease in defect levels and shift in Raman modes of annealed VAZO nanorod samples. In a nutshell, this research on VAZO nanorods would be helpful for fabrication of optoelectronic devices with improved efficiency.

## Figures and Tables

**Table 1:** Average diameter and lengths of VAZO nanorods grown by PLD technique.

Serial No.	Number of Shots	Temperature	Average Diameter of the Nanorods	Average Length of the Nanorods	Standard Deviation (Diameter of Nanorods)	Standard Deviation (Length of Nanorods)	Average Aspect Ratio
1	5000	500 °C	79 nm	286 nm	2.5 nm	2.35 nm	3.43
2	5000	550 °C	185 nm	900 nm	2.6 nm	0.57 µm	8.44
3	10,000	550 °C	162 nm	1 µm	1.6 nm	0.36 µm	7.72

**Table 2:** XRD Peak positions of (0002) plane of ZnO nanorods deposited on Si substrate.

Serial No.	Number of Pulsed Laser Shots	Temperature (°C)	FWHM (Degree) (0002)	Interplanar Spacing (d) (Å)	*c* (Å)	*a* (Å)	$\frac{c}{a}$
1	5000	500	0.268	2.583	5.166	3.31	1.56
2	5000	550	0.268	2.612	5.224	3.23	1.61
3	10,000	550	0.258	2.572	5.144	3.22	1.59
4	15,000	550	0.287	2.570	5.140	3.11	1.65
5	5000	600	0.242	2.577	5.154	3.22	1.60

**Table 3:** Raman peak E_2_ high mode positions of VAZO nanorods.

Serial. No	Number of Shots	Temperature (°C)	E_2_ High (cm^−1^) (Peak Position)	E_2_ High (cm^−1^) (FWHM)
1	5000	500	436.1	16.5
2	5000	550	436.2	28.1
3	10,000	550	435.7	21.1
4	15,000	550	435.7	24.6
5	5000	600	431.94	18.8
6	5000 (annealed in O_2_)	500	431.36	22.9
7	5000 (annealed in H_2_)	500	431.05	24.6

**Table 4:** Defect peak positions of VAZO nanorods from PL spectra.

Serial. No	Number of Shots	Temperature (°C)	Defect Peak Position (eV)
1	5000	500	2.08
2	5000	550	2.23
3	10,000	550	2.18
4	15,000	550	2.01
5	5000	600	2.08
6	5000 (annealed in O_2_)	500	2.13
7	5000 (annealed in H_2_)	500	2.14
